# Ischaemia reperfusion injury: mechanisms of progression to chronic graft dysfunction

**DOI:** 10.1007/s00467-018-3940-4

**Published:** 2018-03-30

**Authors:** Gerhard R. Situmorang, Neil S. Sheerin

**Affiliations:** 1 Institute of Cellular Medicine, Newcastle University, Newcastle upon Tyne, NE2 4HH UK; 2 Urology Department, Faculty of Medicine Universitas Indonesia - Cipto Mangunkusumo Hospital, Jakarta, 10430 Indonesia

**Keywords:** Kidney transplantation, Acute ischaemic injury, Delayed graft function, Chronic graft dysfunction, HIF-1, Hypoxia, Endothelial dysfunction

## Abstract

The increasing use of extended criteria organs to meet the demand for kidney transplantation raises an important question of how the severity of early ischaemic injury influences long-term outcomes. Significant acute ischaemic kidney injury is associated with delayed graft function, increased immune-associated events and, ultimately, earlier deterioration of graft function. A comprehensive understanding of immediate molecular events that ensue post-ischaemia and their potential long-term consequences are key to the discovery of novel therapeutic targets. Acute ischaemic injury primarily affects tubular structure and function. Depending on the severity and persistence of the insult, this may resolve completely, leading to restoration of normal function, or be sustained, resulting in persistent renal impairment and progressive functional loss. Long-term effects of acute renal ischaemia are mediated by several mechanisms including hypoxia, HIF-1 activation, endothelial dysfunction leading to vascular rarefaction, sustained pro-inflammatory stimuli involving innate and adaptive immune responses, failure of tubular cells to recover and epigenetic changes. This review describes the biological relevance and interaction of these mechanisms based on currently available evidence.

## Introduction

The global burden of chronic kidney disease (CKD) has been steadily increasing over recent years. This has resulted in a continuing rise in the number of kidney transplants performed, but also identifies a major problem for kidney transplant programmes—a shortage of available donors. The increasing use of extended criteria donors and utilisation of ex vivo normothermic perfusion technologies are some of the steps taken to increase the donor pool. However, this will also increase the number of organs with more severe ischaemic injury, potentially increasing the risk of delayed graft function and earlier deterioration in graft function. A comprehensive understanding of molecular events involved in post-ischaemic kidney injury and how they may affect the long-term function of the organ is crucial in formulating prevention strategies and to identify novel therapeutic targets. We review the available evidence on the underlying mechanisms involved in the progression of acute kidney injury (AKI) to CKD, focusing on transplantation.

### The link between AKI and CKD

**Fig. 1:**
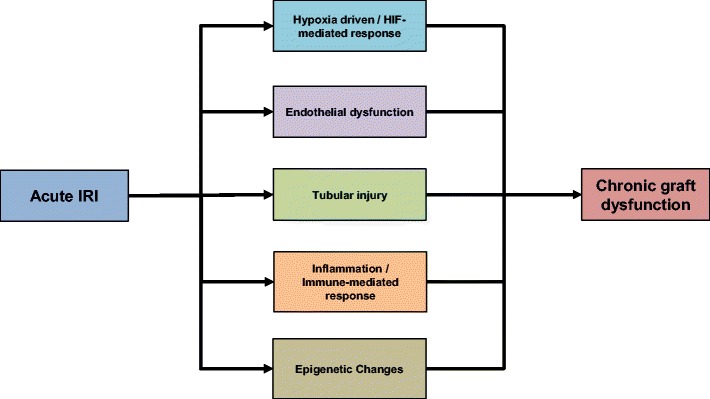
Mechanisms proposed in the development of chronic graft dysfunction. IRI ischaemia-reperfusion injury

An increasing number of clinical epidemiology studies have reported an association between AKI and the development of CKD [1, 2]. Despite the well-established epidemiological link between AKI and CKD, evidence to support a causative relationship between the two clinical entities is still lacking. Transplant patients serve as an ideal cohort to investigate the link between acute ischaemic injury and development of late organ dysfunction. Ischaemic injury in kidney transplantation initially manifests as delayed graft function (DGF), which is associated with prolonged hospitalisation and the need of renal replacement therapy post-transplant. Nevertheless, recovery of organ function is usually achieved in patients with DGF, indicating a degree of resolution of acute ischaemic injury. However, several studies have reported that patients with DGF have increased risk of acute rejection and poorer long-term renal function [3]. These findings provide a link between acute ischaemic injury to the kidney and long-term deterioration in graft function, despite relatively “normal” function in the earlier stages. Several mechanisms have been proposed in the development of chronic dysfunction, as illustrated in Fig. 1. It is important to consider these mechanisms not as separate pathophysiologic entities, rather as components of an intricate network with many overlapping, co-existing pathways.

## Role of hypoxia and hypoxia-inducible factor (HIF)

### The renal response to hypoxia

The anatomy of the nephron and renal microcirculation plays a crucial role in understanding the effect of ischaemia on the kidney. In physiological conditions, kidneys receive approximately 20% of cardiac output. This blood flow is primarily channelled to the cortex with blood flow to the medulla predominantly from the vasa recta, a continuation of efferent arterioles of the juxtamedullary glomeruli. In comparison to other parts of the body, the kidney, particularly the outer medulla, operates at lower oxygen tensions, both during normoxia and hypoxia [4]. This is due to post-glomerular arterio-venous shunting and high oxygen demands [5]. In response to hypoxia, kidney blood flow may alter dramatically, especially to the outer medullary region, reducing oxygen delivery and increasing hypoxic injury in this region.

The capacity of a kidney to withstand an ischaemic injury or to undergo repair after an ischaemic injury is highly dependent upon the available nephron mass, pre-existing glomerulosclerosis and arteriosclerosis [6, 7], features typically associated with increasing donor age. In experimental models of transplantation, older kidneys were more susceptible to ischaemic injury even after a brief ischaemia time [6]. A retrospective analysis of the Australian and New Zealand transplant registry by Wong et al. [8] also suggests a significant interaction between total ischaemic time, donor age and graft function, with higher DGF rates in recipients receiving kidneys from older donors.

The mechanism by which a further reduction in oxygen delivery to the kidney induces AKI has been well established. However, demonstrating the role of hypoxia in progression of kidney injury in the longer-term is more challenging. Several studies have shown an association between chronic tubulointerstitial hypoxia, oxidative stress and chronic inflammation, and that these factors are involved in the progression of CKD [9, 10].

### The role of hypoxia inducible factor

**Fig. 2:**
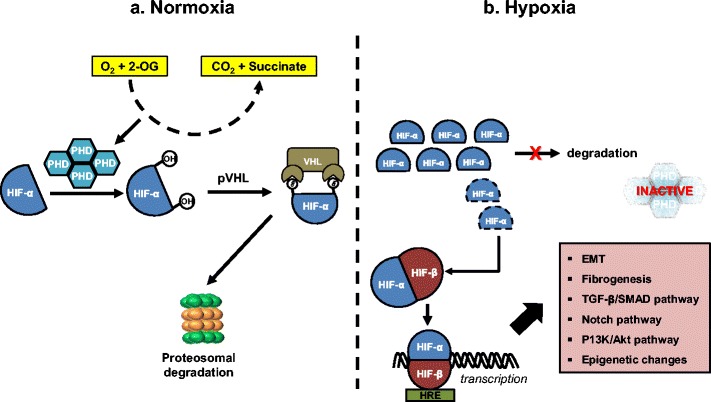
HIF-α canonical pathway during **a** normoxia and **b** hypoxia

Cellular adaptation to hypoxia is largely regulated by a heterodimeric transcription factor, hypoxia-inducible factor (HIF), which consists of two sub-units; α and β. Whereas the β sub-unit is relatively insensitive to alteration in oxygen levels, the level of the alpha sub-unit (HIF-α) is highly dependent on cellular oxygen tension [11, 12]. HIF-α has two major isoforms; HIF-1α, HIF-2α and one additional minor isoform HIF-3α [11, 13]. HIF-α is expressed at a basal level in cells but in normoxic conditions HIF-α is hydroxylated by HIF-prolyl hydroxylase (PHD) leading to ubiquitination and degradation. In order to perform their catalytic function, HIF-prolyl hydroxylases require O_2_, Fe and 2-oxoglutarate (an intermediate in the Tri-carboxylic acid cycle) [14]. In normoxic conditions, degradation occurs very rapidly, resulting in very short half-life of HIF-α, making it almost undetectable in healthy cells. During hypoxia, HIF-propyl hydroxylase function is reduced, leading to accumulation of HIF-α in the cytoplasm. HIF-α forms a complex with the constitutively expressed HIF-β, leading to nuclear translocation and binding of the complex to hypoxia-response elements (HRE), initiating gene transcription (see Fig. 2).

Conde et al. found that HIF-1α is expressed during ischaemia, but disappears 24 h after hypoxia is reversed, then reappears in late reperfusion [15], suggesting recurrence of tissue hypoxia during the cell regenerative phase [16]. In human kidney allograft biopsies, upregulation of HIF-1α is detected immediately after engraftment, at 10–14 days post procedure, but not after 3 months [17]. These findings suggest that renal hypoxia occurs not only during the acute phase of ischaemic injury but also during the recovery phase, presumably due to the activity of oxygen-consuming regenerative processes.

### HIF activation and fibrogenesis: antagonist or protagonist

Current evidence is conflicting regarding the role of HIF in progression of CKD. Experiments using cobalt chloride (CoCl_2_) and dimethyloxalyglycine (DMGO) to stabilise HIF and increase expression of HIF target genes in an ablation/infarction mouse model showed up-regulation of vascular endothelial growth factor (VEGF), glucose transporter 1 (GLUT1) and cell proliferation, indicating a reno-protective effect of HIF [18]. Another study in mice assessed the relationship between HIF-1, ischaemic acute kidney injury (AKI) and the development of fibrosis by increasing HIF level using pre-ischaemic pharmacological inhibition of HIF-propyl hydroxylases. Increased HIF level was associated with reduced fibrosis and less alpha-smooth muscle actin (α-SMA) expression at 21-day post ischaemia reperfusion injury (IRI) [19]. This anti-fibrotic effect was not observed when HIF-propyl hydroxylase inhibition was given after IRI. In addition, Kobayashi et al. documented reduced fibrosis in mice subjected to unilateral ureteral obstruction (UUO) and global activation of HIF [20].

In contrast to these observations, there is a significant body of evidence to support a pro-fibrotic role of HIF. Wang et al. showed that silencing HIF-1α in a rat model of chronic renal ischaemia decreased collagen and α-SMA induction [21]. Moreover, HIF-1α knockout prevented transforming growth factor-β1 (TGF-β1) induced epithelial-to-mesenchymal transition (EMT) in mouse proximal tubular epithelial cells following unilateral ureteral obstruction (UUO) [22]. Our unpublished data shows that hypoxia or stabilisation of HIF-1α is associated with inhibition of SMAD7 and increased in SMAD3 activity, also suggesting a pro-fibrotic role of HIF. Post-ischaemic renal fibrosis can occur through several mechanisms, including direct transcriptional regulation of pro-fibrotic genes, epithelial-to-mesenchymal transition (EMT) and induction of epigenetic changes. All of these mechanisms can be driven by HIF activation, either through direct regulation or indirectly, involving crosstalk with multiple signalling pathways [23, 24], as discussed in the subsequent sections.

### Direct transcriptional regulation of pro-fibrotic genes by HIF

HIF binding to HREs in gene promotor regions allows direct transcriptional regulation of many genes essential in promoting renal fibrosis. As examples, in a mouse glomerulosclerosis model, Baumann et al. has shown HIF-1α binding to HREs in the promotor region of the collagen type-1 alpha 2 chain (COL1A2) gene [25]. Nuclear accumulation of HIF-1α induced by ischaemia was shown to promote extracellular matrix deposition by renal proximal tubular epithelial cells by direct regulation of plasminogen activator inhibitor-1 (PAI-1) [26].

**Fig. 3:**
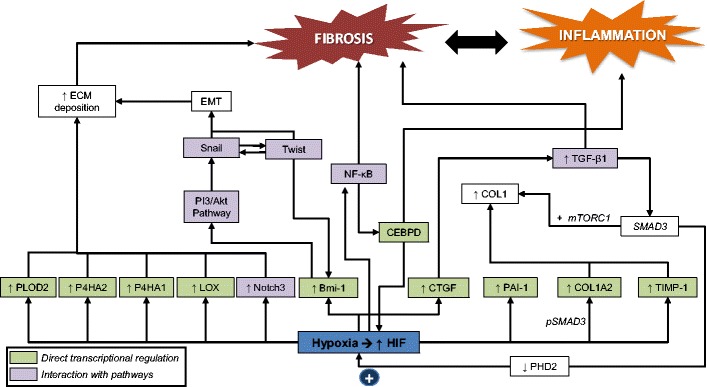
Contribution of HIF-α to the development of post-ischaemic fibrosis

Exposure of human renal fibroblasts to hypoxia in vitro led to significant increase in Col-1 and tissue inhibitor of metalloproteinase (TIMP)-1 production, accompanied with decreased expression of collagenase [27]. Although hypoxia alone induced TGF-β1, the introduction of an inhibitory anti-TGF-β1 antibody had no effect in preventing hypoxia-induced Col-1 and TIMP-1 mRNA expression, suggesting a direct effect of HIF-1α on gene transcription [27]. In addition, a study on human skin fibroblasts demonstrated the contribution of HIF-1α to the development of post-ischaemic fibrosis by directly regulating pro-Collagen prolyl (P4HA1 and P4HA2) and lysyl (PLOD2) genes essential for collagen deposition, extracellular matrix stiffening and collagen fibre alignment (see Fig. 3) [28].

### HIF interaction with the TGF-β pathway

TGF-β is one of the main mediators of renal fibrosis. A relationship between HIF and TGF- β was shown by the increasing level of TGF- β1 produced by tubular cells in response to hypoxia [29]. HIF has been shown to directly regulate the transcription of many genes involved in the TGF- β pathway. In a glomerulosclerosis model, HIF-1α was shown to form a complex with phophoSMAD3 at the COL1A2 promoter, inducing Col-1 synthesis [25]. Similar synergistic interaction of HIF and TGF-β1/SMAD3 signalling has also been reported in the regulation of VEGF, endothelin and erythropoietin expression [24]. Furthermore, accumulation of HIF in the endothelial cells of endothelial specific propyl hydroxylase-2 knockout mice upregulated TGF-β1 expression, resulting in significantly poorer renal function [30]. In a study using a renal tubular cell line in which transcription of HIF-α was absent, there was a decrease in basal and TGF-β1 stimulated Col-1 expression [31]. Altogether, these findings highlight the close interaction between HIF and TGF-β pathway.

Despite being implicated in promoting fibrosis, TGF-β has also been shown to have an immunosuppressive effect, which may be beneficial in a kidney transplant setting. Yoshimura et al. proposed several mechanisms by which TGF-β inhibits the immune response [32]. These mechanisms include suppression of helper T cell differentiation, suppression of T cell activation and proliferation, suppression in macrophages, dendritic cells (DCs) and natural killer (NC) cells, conversion of naive T cells to regulatory T cells, and inhibition of cytokine production (IL-2, IL-4). The effect of the interaction between HIF and TGF-β pathway on the recipients’ immune status, and how this may affect long-term allograft function is not known. However, profiling of biopsy tissues from chronic allograft nephropathy (CAN) patients showed significantly higher expression of both HIF-1α and TGF-β1 compared to the group without CAN [33].

### HIF interaction with other pro-fibrotic pathways

Post-ischaemic induction of HIF also plays a pro-fibrotic role by indirect crosstalk with other pro-fibrotic pathways including Notch, NF-κB and PI3K/Akt pathways [23]. The interaction between HIF and these other pathways is complex (see Fig. 3). HIF can increase the transcription of proteins involved in these other pathways [30, 34]. HIF can also interact with other transcription factors, augmenting their transcriptional activity [35]. In addition, these other pathways can increase HIF gene expression, creating a positive feedback loop [35]. Recently, a transcription factor CCAAT/enhancer-binding protein δ (CEBPD) was discovered as a potential link between hypoxia and inflammation. CEBPD is known to be rapidly induced by inflammatory cytokines, such as IL-1β, in a NF-κB dependent manner. In both an acute and chronic murine model of renal hypoxia, CEBPD was induced in the nuclei of tubular epithelial cells and by direct promoter binding increased HIF transcription and activity [36].

## Endothelial injury and vascular rarefaction

Depending on its severity, ischaemia alone may cause endothelial injury/dysfunction. As a consequence, the endothelium is no longer able to serve as an adequate barrier between the insterstitium and the vascular compartment, loses its ability to control adhesion and infiltration of immune/inflammatory cells and fails to regulate key haemostatic mechanisms [37]. Endothelial cells contribute to progression of IRI by two main mechanisms; (1) increased permeability and (2) vasomotor dysregulation.

Increased endothelial permeability can be attributed to direct injury to the endothelial cells, alterations to the actin cytoskeleton, loss of cell-to-cell junctions and enhanced leukocyte-endothelial interactions [38, 39]. In normal conditions, the endothelium is maintained in a monolayer by the formation of intercellular junctional complexes. These junctional complexes interact with the cell cytoskeleton and other intracellular proteins, and this interaction is highly sensitive to physiological/pathophysiological stimuli, such as ROS, cytokines, lipid mediators and proteases [37]. Release of pro-inflammatory mediators and ROS during IRI will induce phosphorylation, internalisation and degradation of these junctional complexes resulting in endothelium structural damage [40].

**Fig. 4:**
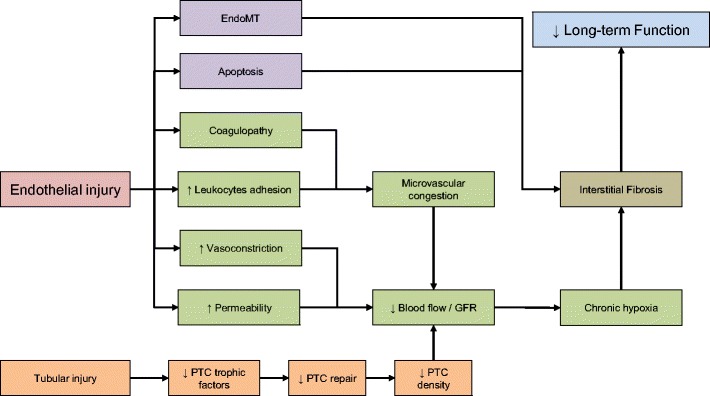
Mechanical obstruction of the capillary network and reduction in blood vessel patency

Increased levels of prostaglandin H2, leukotrienes C4 and D4, increased sympathetic activity and reduced nitric acid synthetase activity have all been documented following endothelial injury, leading to vasoconstriction. [41]. The injured endothelium will also release chemotactic cytokines, increasing leukocytes-endothelial adhesion and release of vasoactive, inflammatory cytokines, which in turn amplify the vasoconstriction that occurs [41]. Exacerbation of hypoxia is closely tied to changes in outer medullary haemodynamic, mainly as a result of IRI induced inflammation, which reduces naturally occurring anti-coagulant activity crucial to prevent micro-coagulapathy [39]. The combination of excessive vasoconstriction, leukocyte activation and subsequent activation of coagulation pathways may lead to mechanical obstruction of the capillary network and reduction in blood vessel patency, which further compromises microcirculatory physiology, especially in the outer renal medulla (see Fig. 4). As a consequence, further ischaemia will ensue, amplifying the initial insult as well as affecting subsequent repair processes.

Significant reduction in peri-tubular capillaries (PTC) density has been suggested as a possible factor that makes the post-ischaemic kidney susceptible to further loss of function. Using the bilateral ischaemia reperfusion rat model, Basile et al. showed a 30–50% permanent reduction in PTCs in the outer medulla despite normal tubular morphology. Ischaemic kidneys subsequently developed tubulointerstitial fibrosis [42]. Similar findings were also reported in a kidney transplant cohort. Loss of PTC during the first 3 months post-transplant was associated with increased interstitial fibrosis, tubular atrophy and reduced renal function [43].

Basile et al. [44] proposed three possible mechanisms on how post-ischaemic PTC loss may lead to the development of long-term fibrosis: (1) exacerbation of pre-existing hypoxia, (2) changes in outer medullary haemodynamics, which is linked to impairment in sodium homeostasis, predisposing the kidney to the development of sodium sensitive hypertension and (3) endothelial-to-mesenchymal transition (EndoMT), which promotes proliferation of new fibroblasts. Acute hypoxia has been linked with the activation of pro-fibrotic pathway, and there is strong evidence available to show that PTC loss increases interstitial hypoxia even after the initial acute hypoxic episode has resolved [45]. Alterations in outer medullary haemodynamics affect tubule-glomerular feedback mechanisms that are involved in the regulation of sodium homeostasis, leading to the development hypertension [46]. EndoMT is a potential source of interstitial fibroblasts after hypoxia. A study on bilateral ischaemic kidney mouse model showed prominent co-existing staining of endothelium (CD31 or cablin) and the mesenchymal marker (S100A4) within 6 h after hypoxia, which was sustained for at least 7 days [47]. The role of EndoMT is still not clearly understood but there in increasing that data suggests it makes a significant contribution, in combination with the loss of endothelial regenerative capacity, to the progression of chronic kidney damage [47, 48].

## The role of inflammation and the immune system

Ischaemia reperfusion injury is a result of various mechanisms, including the host inflammatory/immune response. The initiation of inflammation occurs during ischaemia, whilst post-ischaemic events, such as ROS generation, amplifies the response. Therefore, the ischaemic kidney is not merely the target of immune activation. Instead, it plays an active role in promoting immune activation. The acute inflammatory component of IRI involves the expression of cell surface adhesion molecules. To evaluate the effect of IRI on the expression of these adhesion molecules, rat renal grafts were cold preserved for 2, 4, 6, 12, 24 and 48 h, before being transplanted into syngeneic recipients [49]. The study revealed that longer duration of cold-ischaemia led to loss of endothelial integrity and increased expression of VCAM-1. Ischaemic grafts also displayed enhanced intra-graft pro-coagulant capacity and a worse tubular necrosis [49]. Unexpectedly, renal function measured by creatinine and urea were similar in all groups. This implies that there are potentially injurious processes occurring after moderate IRI that are clinically undetectable. Whether the same is true in patients receiving allografts from marginal donors but who do not develop DGF remains an important research question.

### The role of neutrophils and macrophages

Neutrophil adhesion to injured endothelial cells is a rapid and important component in the initiation of damage in the ischaemic kidney. Neutrophils have been shown to migrate into the transplanted organ within 6 h of reperfusion and are attracted by a set of chemokines, including CXCL8 (IL-8), CXCL10, RANTES, IL-17 and MCP-I [50–53]. Damaged cells will be killed by neutrophils by direct phagocytosis or degranulation, releasing proteases, myeloperoxidase, nitrogen species, antimicrobial peptides and cytokines, which further contribute to the generation of ROS [54]. Recruitment of neutrophils also involves endothelial cell expression ICAM1, E and P selectin, which cross-talk with integrins and L-selectin on neutrophils [51, 55, 56]. Inhibiting the accumulation of neutrophils in the kidney may prevent acute kidney injury [53, 56–58]. In contrast, other studies have failed to reproduce beneficial effects of neutrophil depletion and suggest neutrophil independent mechanism in the pathophysiology of acute tubular injury [59, 60]. Nevertheless, the majority of evidence supports a role for neutrophils in the development of post-ischaemic injury, by mechanisms including obstruction of renal microvasculature and release of free radicals and proteases [61].

Several studies have observed a decrease in IRI severity after macrophages depletion prior to injury [62, 63] indicting a role for macrophages in promoting tubular injury during the initial phase of IRI. However, suppressing macrophage function during the repair process has been shown to suppress tubular proliferation, thus impairing the normal recovery process. Therefore, the role of macrophages in renal response to IRI is complex. Pre-clinical studies have described the involvement of macrophages in the early inflammatory response, during cellular regeneration and tissue repair as well as during the development of fibrosis. These diverse roles are played by different sub-types of macrophages based on their activation and functional states. Classical activation of macrophages typically involves interferon gamma (IFNγ). Ischaemia-induced cellular injury also produces danger-associated molecular patterns (DAMPs), which will be recognised by pattern recognition receptors (PRRs) and contribute to classical macrophage activation. These classically activated M1 macrophages are pro-inflammatory and associated with tissue damage. However, they also play an important role in clearing apoptotic cells and debris, thereby initiating repair process [64]. Alternatively activated macrophages include M2a macrophages, which are responsible for wound healing and M2b macrophages, also known as immunoregulatory macrophages. M2a macrophages are activated through IL-4/IL-13 binding to IL-4 receptor, which leads to production of growth factors, collagen precursor synthesis and generation of extracellular matrix. M2b macrophages regulate inflammatory response by producing of immunosuppressive cytokines, IL-10 and TGF-β. Production of TGF-β limits inflammation, but at the same time may contribute to activation of pro-fibrotic pathways. When injury persists, chemokines, macrophage colony-stimulating factors (M-CSF) and IL-34 are secreted to sustain recruitment and retention of macrophages [65]. Blockade of the M-CSF receptor has a protective effect following experimental transplantation [66]. Retention of M2b macrophages in the injured tissue will produce macrophage-derived factors, which subsequently activate and support myofibroblasts, inducing extracellular matrix deposition and fibrosis. The signals responsible for retaining pro-fibrotic macrophages in the kidney remain unclear, but studies using unilateral ureteral obstructive (UUO) rodent model suggest a role for the chemokine receptors CCR1, CCR2, CX3CR1 [65].

### The role of the complement system

The complement system has been well identified as an important, early mediator of the post-ischaemic inflammatory response [67, 68]. Activation of complement is an important factor in the progression of renal disease, and targeting complement has been an attractive therapeutic option due to its involvement in both innate and adaptive immune response to IRI [69, 70]. Ischaemic insult to the kidney has been shown to involve the anaphylotoxins, C3a and C5a, acting through their respective receptors (C3aR and C5aR). Stimulation of C3a/C5a receptors during IRI was shown to increase pro-inflammatory cytokine/chemokine production and tubular injury. The membrane attack complex C5b-9 has also been shown to contribute to the progression of renal damage [71]. Recently, C-type lectin collectin-11 (CL-11/Colec11) was discovered as an activator of mannan-binding lectin (MBL) pathway in the kidney in response to ischaemia. CL-11 acts by recognising L-fucose on kidney tubules following ischaemia [72]. The study showed that global or kidney-specific deficiency of CL-11 reduces post-ischaemic tubular injury and functional loss [72].

### The role of natural killer cells, dendritic cells and lymphocytes

Substantial evidence is available to link natural killer (NK) and NKT cells, renal dendritic cells (DCs), T cells and B cells to early IRI, mainly linking their actions to direct targeting of injured tubular and endothelial cells, activation of neutrophils and macrophages, and secretion of pro-inflammatory cytokines, such as IFN-γ, TNF-α, IL-4 and IL-10. If NK and NKT cell function is reduced, the severity of renal injury following IRI is also reduced [73–75]. DCs contribute to post-ischaemic kidney injury by the secretion of TNF [76]. Biopsies taken from DGF patients suggested an association between DGF and acute rejection due to an imbalance between myeloid DCs (involved in graft rejection) and plasmacytoid DCs (which may play role in graft tolerance) [77].

Although initially regarded as by-standers, current evidence suggests an active role for T cells in the pathogenesis of IRI. Depletion of CD4 and CD8 T cells in murine IRI has been shown to improve renal function and reduce neutrophil infiltration and tubular atrophy [78]. Furthermore, reconstitution of T cells in T cell-deficient mice restores injury to the level seen in normal mice [79]. Depletion of either αβ or γδ T-cells in mice was associated with reduction in renal injury [80]. Nevertheless, a subset of T cells is also recognised to play a role in preventing injury and promoting repair. Pre- and post-ischaemic adoptive transfer of regulatory T cells has been shown to protect the kidney from ischaemic injury, reduce TNF-α and IFN-γ production and accelerate repair [81, 82].

B cells are involved in the adaptive immune response to IRI. To date, studies have shown a predominantly harmful effect of post-ischaemic B cell activation [83]. Depleting B cell in mice was shown to improve renal function and reduce tubular injury after ischaemia [84]. A study by Jang et al. documented that B cell-deficient mice subjected to ischaemia showed more tubular proliferation, less tubular atrophy and higher expression of IL-10 and VEGF [85]. Adoptive transfer of B cells into these mice blocked this effect, suggesting that B cells may interfere with post-ischaemic repair processes. In contrast, another study found worse post-ischaemic renal injury in mice lacking all mature B cells [86], suggesting a more complex and divergent role for B cells in the progression of IRI.

## Tubular recovery and maladaptive repair

The proximal tubule is the main site of injury in acute ischaemic kidney. Consequently, the severity and recurrence of injury at this site acts as an important factor in determining reversibility of the damage and progression to long-term organ failure. Severe and repeated injuries induce worse interstitial fibrosis, distal tubular injury, glomerulosclerosis and atubular glomeruli [87]. Ischaemic tubular injury is most evidently found in the S3 segment of the proximal tubule and initially results in loss of cytoskeletal integrity [88]. The degree of cytoskeletal alteration depends on the severity and duration of ischaemia. This loss of cytoskeletal integrity further modifies cellular polarity, cell-to-cell interactions as well as cell-to-matrix interactions, and loss of function [89].

**Fig. 5:**
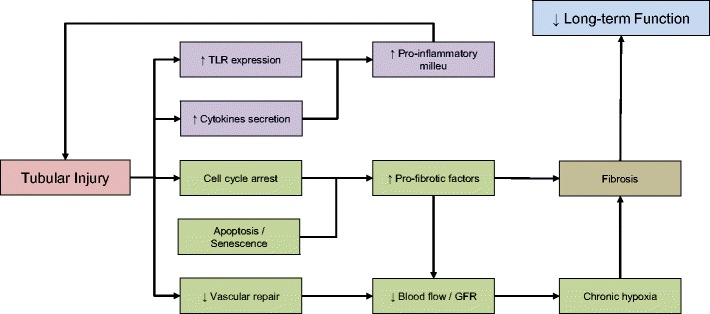
Kidney tubular epithelial cells playing an active role in progression of post-ischaemic tissue damage through several mechanisms

Kidney tubular epithelial cells have been shown to play an active role in progression of post-ischaemic tissue damage through several mechanisms (see Fig. 5). There is substantial evidence that TECs release pro-inflammatory and chemotactic cytokines in response to IRI [89–91], which includes TNF-a, IL-6, IL-1B and TGF-B in addition to chemokines, such as MCP-1, IL-8, RANTES and ENA-78 [90]. This leads to recruitment of immune cells, important for subsequent repair following IRI but also to the damage that occurs. In addition, damaged epithelial cells produce DAMPs, which act as warning signals by activating a series of Toll-like receptors (TLR2, TLR3 and TLR4) and express complement receptors and other co-stimulatory molecules which regulate T lymphocyte activity [41, 92, 93]. Downregulating the expression of TLR-2 on kidney parenchymal cells was shown to reduce the level of pro-inflammatory cytokines (IL-1β, IL-6, MCP-1 and Keratinocyte Chemoattractant) produced by the kidney, thus providing functional and structural protection against IRI [92]. Wu et al. demonstrated upregulation of TLR4 post IRI in TECs and inhibiting TLR4 reduced the severity of IRI [93]. Furthermore, TLR4 knockout mice showed reduced tubular injury with better preservation of renal function after induction of IRI compared with wild type mice [94].

Dedifferentiation of proximal tubule cells caused by IRI may not always be followed by complete re-differentiation and resolution of injury. Rodent kidneys subjected to IRI still had a proportion of abnormal tubules with flat epithelium without brush borders after 14 days. These cells were morphologically abnormal, atrophic and growth arrested. As well as showing strong TGF-β signalling, these abnormal cells also showed persistent loss of phosphate and tension homologue (PTEN) associated with increased expression of vimentin, pro-fibrotic c-Jun N-terminal kinase (JNK) activation and platelet-derived growth factor (PDGF)-B production [95].

TEC secretion of cytokines and growth factors is important for cell survival and repair, however, this should halt once complete regeneration is achieved [96, 97]. A study of five different mouse models of acute kidney injury revealed large numbers of proximal tubule were arrested in the G2/M phase of the cell cycle, which is associated with persistent activation of JNK signalling and higher production of *COL4A1* and *ACTA2* mRNA levels [98]. A number of studies by Venkatachalam et al. also showed that tubular cell arrest and atrophy is linked with increased secretion of fibrogenic peptides, which accelerates proliferation of interstitial pericytes/fibroblasts through multiple pathways, including PI3K-Akt-mTOR, ERK-MAPK, JNK-MAPK and TGF-β pathways [96, 99, 100], eventually resulting in nephron loss. Based on these finding, several authors have investigated the potential usage of cell cycle arrest biomarkers in the detecting acute kidney injury [101].

Tubular cells maladaptive repair is also associated with activation of several other pro-fibrotic pathways, such as Notch and Wnt signalling. Recent work using a mouse model with inducible proximal tubule Wnt1 secretion displayed interstitial myofibroblast activation and proliferation and increased matrix protein production [102]. Interestingly, no evidence of inflammatory cytokine expression, leukocyte infiltration or epithelial injury were detected in these fibrotic kidneys, demonstrating direct paracrine Wnt1 activity in initiating interstitial fibrosis through tubulointerstitial crosstalk [102].

## Epigenetic changes

Recent findings highlight the pivotal role of epigenetic changes caused by acute IRI in damage progression resulting in renal fibrosis and long-term deterioration of function. Hypoxia has been proven to induce epigenetic changes in the form of DNA methylation, histone modification, alteration in chromosome conformation, differentially expressed long non-coding RNAs (lncRNAs) and microRNAs (miRNAs) [103, 104]. Affected cells have been shown to store these changes in form of “memory” [103, 105]. In respect to IRI, this “hypoxic memory” may sustain initial pathological alteration in homeostasis or induce new changes that result in transition from acute to chronic kidney injury. Animal models of acute kidney injury have demonstrated that “hypoxic memory” can promote pro-inflammatory and pro-fibrotic gene expression, such as monocyte chemoattractant protein-1, TGF-β1, and collagen [103]. The development of renal fibrosis has been associated with several DNA methylation and histone modifications. Comparison of the methylation profile of fibroblasts derived from fibrotic and non-fibrotic kidneys showed distinct methylation patterns, including hypermethylation of RAS protein activator-like 1 gene [106]. In UUO model, TGF-β has been shown to increase histone H3 lysine methylation, which increased expression of extracellular matrix gene connective tissue growth factor, Collagen-1 and plasminogen activator inhibitor-1 in mesangial cells. The study also showed inhibition of renal fibroblast accumulation through blocking of class I histone deacetylates [107, 108]. Moderate IRI has been shown to increase TGF-β1 and CTGF protein production, which in turn initiated epigenetic changes in fibroblasts [98]. Bechtel et al. documented hypermethylation of *RASAL1* gene loci [106], which in turn persistently activated Ras, resulting in transformation of fibroblasts to myofibroblasts secreting collagen in a growth-factor independent manner [109]. As described in a previous section of this review, long-term effect of acute IRI is partly mediated by EMT. A cell model of TGF-β-mediated EMT revealed global altered methylation of several heterochromatins, highlighting the role of epigenetic changes in the pathogenesis of IRI progression [110].

Extensive investigations have been made to link miRNA with renal pathologies, such as acute kidney injury, fibrosis, polycystic kidney and neoplasm. In the kidney transplant setting, miRNA expression has been profiled in association with rejection, interstitial fibrosis, tubular atrophy as well as ischaemia and reperfusion injury. In the context of IRI progression to fibrosis, several miRNAs have been examined, among many are miR-363, miR-192, miR-200, miR-21-, miR-34a, miR-155 and miR-127 [111–113]. Our unpublished data shows that the up-regulation of miR-21 that occurs following IRI inhibits SMAD7 activity, contributing to exaggerated tubular cell responses to TGF-β1 and upregulation of pro-fibrotic markers (α-SMA, collagen type-1) and downregulation of E-cadherin.

## Concluding remarks

**Fig. 6:**
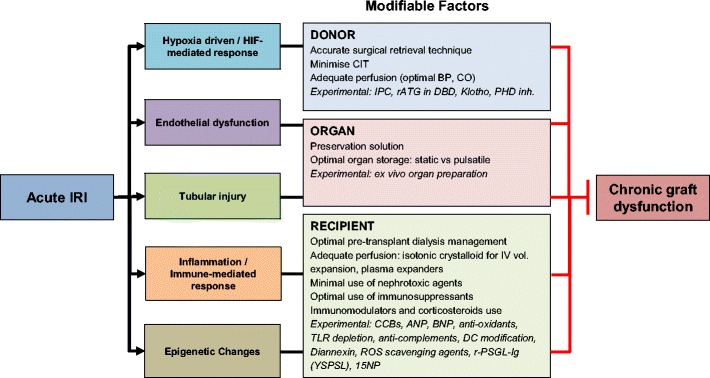
Factors that can be modified to prevent the progression of acute IRI to chronic graft dysfunction. IRI ischaemia-reperfusion injury

We have reviewed currently available evidence on potential mechanisms of acute ischaemic renal injury progression to long-term organ dysfunction. Despite the lack of evidence to prove a causative association between AKI and CKD, strong epidemiological correlates and substantial biological mechanistic links clearly point to the impact of early post-ischaemic events on the development of long-term graft dysfunction. Biological links that connect acute IRI to chronic dysfunction include; (1) HIF-1α driven changes, (2) endothelial and epithelial injury that leads to cellular senescence and maladaptive repair, (3) inflammation/immune system driven processes and (4) epigenetic alteration, all of which may lead to chronic hypoxia and fibrosis as the main underlying pathophysiology. The severity and frequency of the initial insult are crucial factors in determining possible occurrence of long-term consequences. It is also important to acknowledge the active roles played by the cells within the kidney. This is especially of importance in kidney transplant setting, as the repair mechanisms following IRI will vary greatly, depend on the characteristics of the donor kidney (donor type, age, ischaemic time, etc.), characteristics of the recipient (age, underlying disease, co-morbidities, etc.) and various perioperative parameters (haemodynamic fluctuations, warm ischaemia time, the use of prophylaxis for IRI, etc.). In addition, unlike native kidneys, the cellular and molecular effects of IRI in the transplanted kidneys are influenced by immunosuppressive agents. This will have an effect in the kidney’s susceptibility to IRI and its capacity for repair. Modification of the contributing factors to IRI through careful donor selection and preventative procedures are essential to prevent long-term consequences of IRI (see Fig. 6). In the era of rising CKD incidence and where extended criteria donor organs are increasingly utilised, our understanding of IRI-induced molecular events is pivotal in the search for interventions to improve organ quality, thus achieving longer graft survival.
